# Development of TaqMan Probe-Based Insulated Isothermal PCR (iiPCR) for Sensitive and Specific On-Site Pathogen Detection

**DOI:** 10.1371/journal.pone.0045278

**Published:** 2012-09-25

**Authors:** Yun-Long Tsai, Hwa-Tang Thomas Wang, Hsiao-Fen Grace Chang, Chuan-Fu Tsai, Ching-Ko Lin, Ping-Hua Teng, Chen Su, Chien-Chung Jeng, Pei-Yu Lee

**Affiliations:** 1 Department of Research and Development, GeneReach Biotechnology Corporation, Taichung, Taiwan; 2 Institute of Nanoscience and Department of Physics, National Chung Hsing University, Taichung, Taiwan; 3 Institute of Medical Biotechnology, Central Taiwan University of Science and Technology, Taichung, Taiwan; University of Kansas Medical Center, United States of America

## Abstract

Insulated isothermal PCR (iiPCR), established on the basis of Ralyeigh-Bénard convection, is a rapid and low-cost platform for nucleic acid amplification. However, the method used for signal detection, namely gel electrophoresis, has limited the application of iiPCR. In this study, TaqMan probe-based iiPCR system was developed to obviate the need of post-amplification processing. This system includes an optical detection module, which was designed and integrated into the iiPCR device to detect fluorescent signals generated by the probe. TaqMan probe-iiPCR assays targeting white spot syndrome virus (WSSV) and infectious myonecrosis virus were developed for preliminary evaluation of this system. Significant elevation of fluorescent signals was detected consistently among positive iiPCR reactions in both assays, correlating with amplicon detection by gel electrophoresis analysis. After condition optimization, a threshold value of S/N (fluorescent intensity_after_/fluorescent intensity_before_) for positive reactions was defined for WSSV TaqMan probe-iiPCR on the basis of 20 blank reactions. WSSV TaqMan probe-iiPCR generated positive S/Ns from as low as 10^1^ copies of standard DNA and lightly infected *Litopenaeus vannamei*. Compared with an OIE-certified nested PCR, WSSV TaqMan probe-iiPCR showed a sensitivity of 100% and a specificity of 96.67% in 120 WSSV-free or lightly infected shrimp samples. Generating positive signals specifically and sensitively, TaqMan probe-iiPCR system has a potential as a low-cost and rapid on-site diagnostics method.

## Introduction

Polymerase chain reaction (PCR), with its high sensitivity and specificity, is a powerful tool for pathogen detection in plants, animals, or humans [1]–[4]. PCR reactions are carried out conventionally in a thermocycler that provides the required temperatures for different cycling steps. Since most of the reaction time is spent on temperature ramping to bring the solutions to the desired temperatures, shortening the ramp time would speed up the whole process. Current models of thermocyclers have mostly achieved relatively high ramp rates by providing efficient heating and cooling through the integration of Peltier element technology. Alternatively, ramp time could be shortened by decreasing heat capacitance of the system, including the heater and reaction volume, by micromachining of these components [5]–[8]. For example, miniaturized PCR chips have been made available by the advent of micro-electro-mechanical-systems technology. However, problems such as high costs of the reaction vessels and instruments, problems in product manufacturing, and sub-optimal detection methods have prevented these methods from being applied to point-of-care diagnostics for resource-poor environment.

Recent studies showed that the Rayleigh-Bénard convective PCR method could generate significant amounts of PCR amplicons within less than 30 min in a simple heating device [9]–[11]. In Rayleigh-Bénard convective PCR, spontaneous fluid convection in a cylindrical vessel is driven by temperature gradients that are formed simply by heating the vessel from the bottom at a fixed temperature. Consequently, reaction constituents are circulated through zones of temperature gradients. In theory, the denaturation, annealing, and extension steps of PCR take place at the bottom, top, and middle zones of the vessel, respectively. Previously, convective PCR in capillary tubes was found to be sensitive to environmental temperature fluctuations and inherently unstable [10]. Relatively consistent PCR amplification was later demonstrated in the insulated isothermal convective PCR (iiPCR) system, which is carried out in the R-tube™ (GeneReach, Taichung, Taiwan) within a thermally baffled device [11], [12]. Notably, iiPCR achieved sensitivity and specificity comparable to those of conventional nested PCR. Requiring a simple and inexpensive incubator and relatively short reaction time, iiPCR method has a potential to become a powerful tool for point-of-care molecular diagnostics. However, analysis of iiPCR products required gel electrophoresis [11], [12], which is time-consuming and makes the method considerably prone to cross contamination.

Various fluorescent dye-based methods, including generic double stranded DNA (dsDNA) intercalating dyes, dual hybridization probes, TaqMan probes, scorpions and molecular beacons, have been developed to allow sensitive real-time signal detection of PCR and eliminate post-amplification processing [13]–[16]. When excited by lights of appropriated wavelengths, generic dsDNA dyes, such as SYBR® green 1, generate elevated levels of fluorescence only when they are associated with dsDNA. However, generic dyes emit signals from both target and non-specific products (NSPs), resulting in a higher false-positive rate without post-reaction melting curve analysis. In contrary, sequence-specific probes produce signals only when target amplicons are made. For example, TaqMan hydrolysis probes, labeled with a reporter dye at one end and a quencher dye at the other, are designed to hybridize specifically with target amplicons at the annealing step. Hydrolysis of the probes by DNA polymerase in the subsequent extension step separates the reporter from the quencher dye, resulting in emission of fluorescent signals from the reporter dye.

In this study, the TaqMan probe technique was integrated with iiPCR for specific detection of target amplicons, as production of NSPs were noted in iiPCR periodically [12]. In parallel, an optical detection module was developed and built into the iiPCR device for direct detection of fluorescent signals derived from the probes. We provided evidences to demonstrate that the TaqMan probe-based iiPCR could produce signals sensitively and specifically and the modified iiPCR device could resolve positive from negative results reliably.

## Materials and Methods

### Design of the Optical Detection Module for TaqMan Probe-based iiPCR Assay

**Figure 1:**
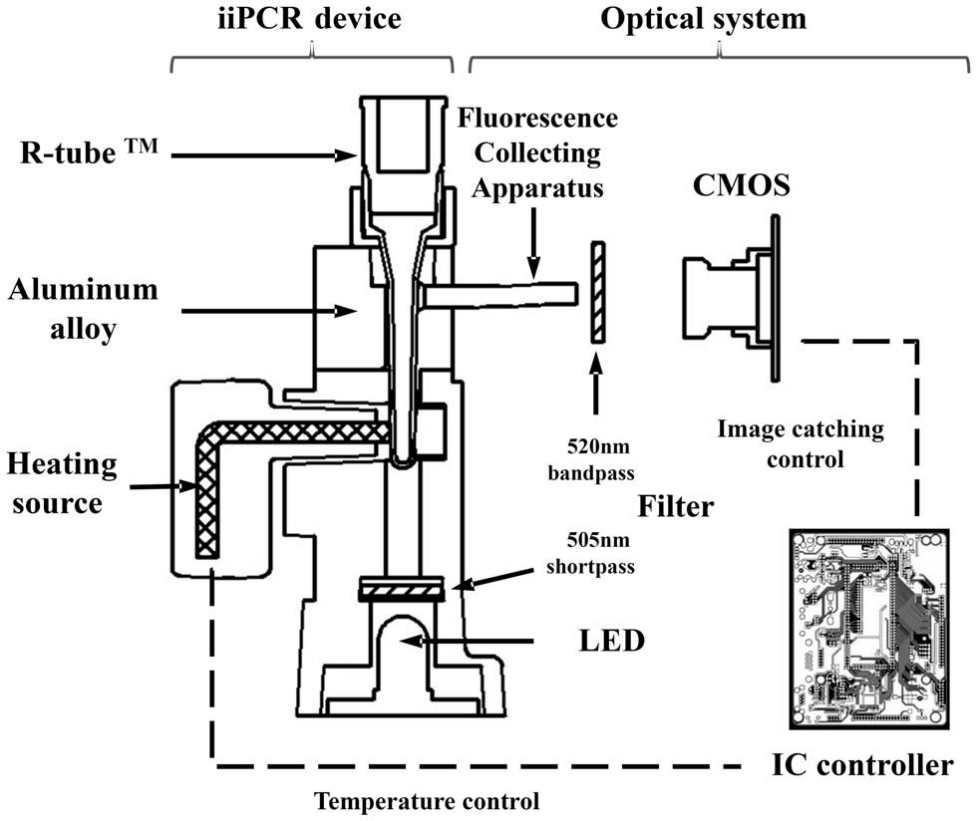
Integration of an optical detection module with the iiPCR module in the modified iiPCR device. The diagram depicts the iiPCR module and the optical detection module, which consists of an LED light source, a 505-nm short-pass filter, a 520-nm bandpass filter, fluorescence collecting apparatus, a CMOS, and an IC controller. The dotted lines indicate that the IC controller modulates the heating source of R-tubes to provide appropriate reaction temperatures and directs CMOS to collect and record light signals before and after a reaction.

An optical detection platform was integrated into the original iiPCR device (Fig. 1) [11] to detect the fluorescent signals. For excitation of 6-FAM™, 465-nm blue LED light was filtered by a 505-nm shortpass filter. From the opposite side of the reaction vessel tube, fluorescent signals were filtered through a 520-nm bandpass filter and collected by an integrated circuits (IC) controller-regulated CMOS sensor. The intensity of the fluorescent signal was determined by averaging the signals from 30 images of each one-second exposure. Signals collected before and after an iiPCR reaction were recorded separately.

### PCR Amplification

TaqMan probes were designed on the basis of the suggestions provided for real-time PCR [17]: TaqMan probes should preferentially have melting temperatures 10°C higher than the primers. The probes are labeled with a reporter dye on the 5′ end and a quencher dye on the 3′ end. The presence of 5′-end guanosine residue and long sequences of identical nucleotides should be avoided.

The 50-µl iiPCR mixture, containing pWSSV1 plasmid [12], 0.5 µM forward primer (5′-AATGGTCCCGTCCTCATCTCA-3′), 0.5 µM reverse primer (5′-GCTGCCTTGCCGGAAATT-3′), 0.15 µM WSSVprobe (5′-6-FAM-TGTGTGATAGACGGCATTCTTCATGGC-BHQ-1-3′) (Sigma-Aldrich, St. Louis, MO, USA), 1X Uni-ii HS Buffer (GeneReach, Taichung, Taiwan), and 25 units of *Taq* DNA polymerase (BioMi, Taichung, Taiwan), was assembled in an R-tube™ (GeneReach) and incubated in the modified iiPCR device for designated periods of time. Amplicons were analyzed subsequently on a 12% polyacrylamide gel in TAE buffer (40 mM Tris, 20 mM acetic acid, 1 mM EDTA) and visualized by ethidium bromide staining. The oligonucleotides for the infectious myonecrosis virus (IMNV) iiPCR assay are forward primer (5′-CACAGCCCACAATCGGTACGTATTAAA-3′), reverse primer (5′-TGTCCCAACCACCCAAATTCAT-3′), and IMNVprobe (5′-6-FAM-TGCACCTGCTACCACTTCT-MGB-NFQ-3′). The pIMNV plasmid, which contains a 340-bp partial sequence of coat protein gene from IMNV genome, was used as the template.

Real-time PCR assays were carried out in an ABI-7500 real time PCR machine (Applied BioSystem, Life Technologies, Carlsbad, CA) using the same DNA standards and reaction mixtures as described for iiPCR. The program included an incubation period at 94°C for 2 min, and 40 cycles of 94°C for 20 sec and 60°C for 1 min. Fluorescence measurements were recorded at the 60°C step.

### Sample Collection and DNA Extraction

*Litopenaeus vannamei* specimense were purchased from local markets. No specific permits were required for the described field studies. Shrimp DNA extracts were prepared by using IQ Plus™ Extraction Kit (GeneReach). Briefly, pleopods (about 20 mg) grounded thoroughly in 500 µl of Solution 1 were combined with 500 µl of Solution 2 and incubated at room temperature for 1 min. After centrifugation at 12,000×g for 1 min, 500 µl of the supernatant were transferred into a spin column and centrifuged at 12,000×g for 1 min. After one wash with 500 µl of Solution 2, the column was centrifuged at 12,000×g for 3 min to remove any residual buffer. Nucleic acids were eluted with 200 µl of Solution 3 and stored at −20°C afterwards. WSSV-positive samples were identified by the IQ2000™ WSSV Detection and Prevention System (IQ2000 WSSV DPS, GeneReach), an OIE-registered commercial kit (World Organization for Animal Health, http://www.oie.int/vcda/eng/envcda registre.htm). Reaction of the IQ2000 WSSV DPS was assembled and carried out as described by the manufacturer. Briefly, the thermo-cycler program for the first PCR started with denaturation at 94°C for two min, followed by 15 cycles of 94°C for 20 sec, 62°C for 20 sec and 72°C for 30 sec, and a cycle of 72°C for 30 sec and 20°C for 20 sec. The second PCR included 30 cycles of 94°C for 20 sec, 62°C for 20 sec and 72°C for 30 sec and one cycle of 72°C for 30 sec and 20°C for 20 sec. PCR products were analyzed by agarose gel electrophoresis.

## Results

### Feasibility Evaluation of TaqMan Probe-based iiPCR

WSSV, a large dsDNA virus, infects a broad range of freshwater and marine crustaceans including shrimp, crabs, crayfish and lobsters [18]–[20]. This study was based on a previously established white spot syndrome virus (WSSV)-specific iiPCR assay [12], which could generate ampiicons from a standard plasmid DNA and diseased samples. Initially, a TaqMan probe was incorporated into WSSV iiPCR to evaluate whether the probe could be hydrolyzed during iiPCR. The WSSVprobe was labeled with 6-carboxyfluorescein (6-FAM™; maximum excitation and emission wavelength, 494 nm and 518 nm, respectively) reporter and Black Hole Quencher (BHQ-1) (maximum excitation 534 nm and no light emission), two commonly used dyes. The reaction was carried out in the original iiPCR device and transferred to regular real-time PCR tubes for fluorescence measurement in an ABI-7500 real time PCR machine (Applied BioSystem) using the filter for 6-FAM™. WSSV probe was added to WSSV iiPCR containing 10^3^ copies of target standard DNA. Hydrolysis of TaqMan probe was evaluated preliminarily by measuring fluorescent signals before and after iiPCR incubation. Compared to basal signals collected before iiPCR, substantial increases in fluorescence intensity were detected after iiPCR (data now shown), suggesting that significant hydrolysis of the TaqMan probe did take place during iiPCR. As preliminary data showed hydrolysis of TaqMan was possible in iiPCR, an optical detection platform (Fig. 1, Materials and Methods) was designed and integrated into the original iiPCR device [11] for easy detection of the signals generated in TaqMan probe-iiPCR.

### Target-specific Fluorescent Signals were Generated in TaqMan Probe-iiPCR and Detected by the Optical System

**Figure 2:**
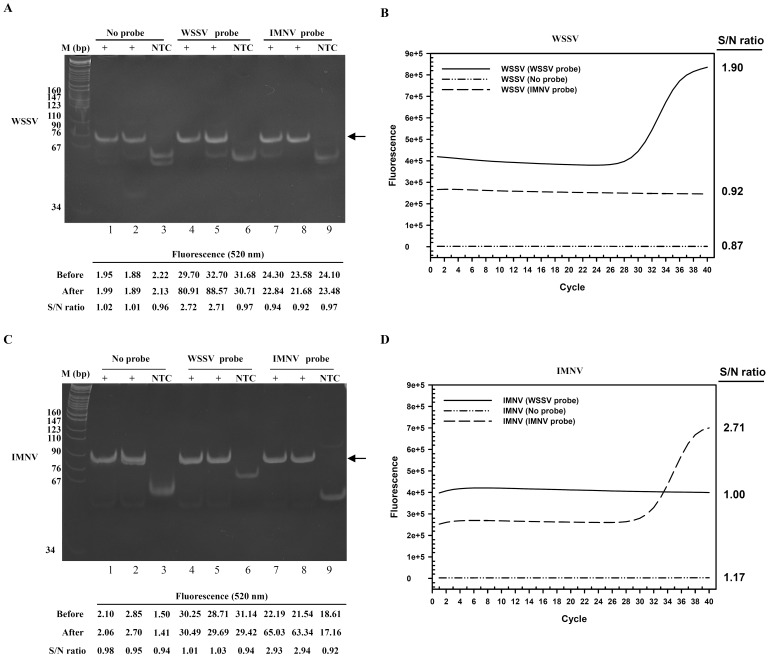
Generation and detection of TaqMan probe hydrolysis in iiPCR. Target pWSSV1 and pIMNV plasmids (10^3^ copies) were subjected to WSSV (A) and IMNV (C) iiPCR, respectively, for 30 min in the presence of WSSVprobe, IMNVprobe, or no probe. Fluorescent signals (520 nm) of individual reactions were collected before and after iiPCR by the modified iiPCR and shown below the gel image. After the reactions were completed, amplicons were also analyzed on a 12% polyacrylamide gel in 1X TAE buffer. WSSV (B) and IMNV (D) real time PCR assays containing the same components as in iiPCR were carried out as described in Materials and Methods. The result shown here is a representative of at least three experiments with similar results. Arrows, iiPCR amplicons; M, DNA size markers; NTC, no template control (ddH_2_O); “before”, fluorescent signals detected before reaction; “after”, fluorescent signals detected after reaction; S/N ratio, signal intensity_after_/signal intensity_before_ for iiPCR; signal intensity_cycle 40_/signal intensity_cycle 1_ for real time PCR.

To evaluate whether the presence of additional oligonucleotides interferes with iiPCR amplification, WSSV iiPCR assays were carried out with or without a TaqMan probe and products were examined by gel electrophoresis analysis. The 71-bp WSSV amplicons were generated from a standard plasmid in the absence (Fig. 2A, lanes 1 and 2) or presence (Fig. 2A, lanes 4 and 5) of 150 nM TaqMan probe. Noticeably, the 520-nm signals collected after WSSV TaqMan probe-iiPCR (after) were significantly higher than those before the reaction (before) (Fig. 2A), supporting that 6-FAM™ signals were produced and could be detected efficiently by the optical system in the modified iiPCR device. Significant probe hydrolysis was detected when the same mixtures were subjected to a real-time PCR program in an ABI 7500 apparatus (Fig. 2B).

To confirm that probe hydrolysis takes place with different targets, IMNV TaqMan probe-iiPCR was designed (see Materials and Methods) and tested, using a plasmid DNA template containing a cDNA fragment derived from genome of IMNV, another important shrimp pathogen. Similarly, an 82-bp amplicon and significant fluorescent signals were produced in the presence of IMNVprobe in IMNV TaqMan probe-iiPCR (Fig. 2C and 2D).

S/N ratios (signal intensity_after_/signal intensity_before_) >2 were detected consistently in iiPCR reactions that included the target TaqMan probe (Fig. 2A and 2C, lanes 4 and 5), indicating that significantly increases of fluorescent signals were resulted by iiPCR within 30 min. Similar trends of S/N ratio changes were observed when the same iiPCR mixtures were subjected to 40 cycles of real-time PCR programs (Fig. 2B and 2D), indicating hydrolysis of the TaqMan probes in iiPCR was similar to that in real-time PCR cycles. Furthermore, S/N values of about 1.00 were detected in NTC controls (Fig. 2A and 2C, lanes 6 and 9) and in reactions that included target template and a non-target probe (Fig. 2A and 2C, lanes 7 and 8), implying that sequence-specific TaqMan probes are hydrolyzed only when the target amplicons are generated. Overall, the results demonstrate that TaqMan probes could be hydrolyzed significantly and specifically during iiPCR amplification and the optical system could detect the fluorescent signals effectively.

### Effects of Probe Concentration on Signal Production in TaqMan Probe-iiPCR

**Figure 3:**
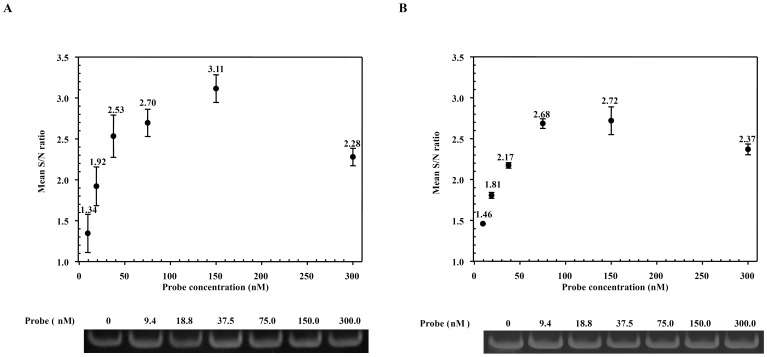
Effects of concentration of TaqMan probe on production of fluorescent signals. Different concentrations (9.4, 18.8, 37.5, 75, 150, or 300 nM) of WSSV probe or IMNV probe were added to the WSSV (A) or IMNV (B) iiPCR, respectively. Each reaction was performed in triplicate. Mean S/N ratio of each reaction (shown on each mark) was plotted against probe concentration. Mean S/N ratios for reactions containing no probe (not shown on the plot) were 0.97 (SD = 0.08) and 0.94 (SD = 0.01) for WSSV and IMNV assays, respectively. Bottom panel shows an example of gel electrophoresis analysis. S/N ratio, fluorescent intensity_after_/fluorescent intensity_before_. Amplicons were analyzed on a 12% polyacrylamide gel in 1X TAE buffer. M, DNA size markers.

Concentration of oligonucleotide is known to influence the outcome of PCR. Different concentrations (0 to 300 nM) of probe were tested in WSSV and IMNV iiPCR to evaluate the effects of probe concentration on target amplification and signal production. Gel electrophoresis analysis showed that significant amounts of amplicons were generated in the presence of 9.4 nM probe in both assays (Fig. 3A and 3B). S/N ratios increased steadily with concentrations of TaqMan probe between 9.4 and 75 nM. Comparable S/N ratios were detected when the samples were subjected to optical analysis in an ABI 7500 real-time PCR machine (Applied BioSystem) before and after iiPCR (data not show). The highest S/N ratios were found around 150 nM and 100–150 nM for the WSSV and IMMNV iiPCR assays, respectively. As similar efficiency in probe hydrolysis was observed in both WSSV and IMNV TaqMan probe-iiPCR, subsequent studies were focused on the development of WSSV assay.

### Optimization of WSSV TaqMan Probe-iiPCR Assay and Definition of Signal Threshold

**Figure 4:**
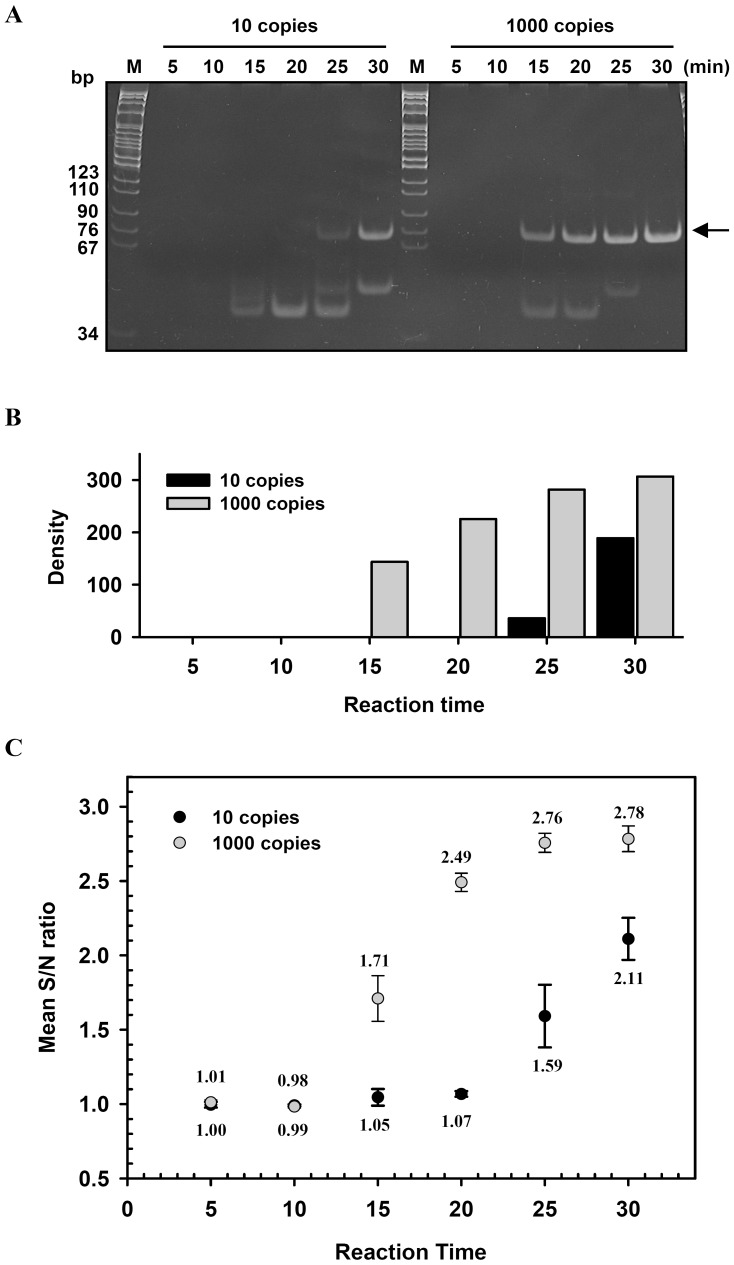
Time course of WSSV TaqMan probe-iiPCR. WSSV TaqMan probe-iiPCR reactions containing 10^1^ and 10^3^ copies of pWSSV1 were allowed to be carried out for 5, 10, 15, 20, 25, or 30 min. Amplicons were detected by 12% polyacrylamide gel analysis (A), and the intensity of the bands was estimated by densitometry (ImageJ program, NCBI) (B). In addition, fluorescent signals of three independent experiments were collected, and S/N ratios and SDs were calculated (C). Arrows, iiPCR amplicons; M, DNA size markers; S/N ratio, fluorescent intensity_after_/fluorescent intensity_before_.

First, time course of signal production in WSSV TaqMan probe-iiPCR assay was evaluated. Reactions spiked with 10^1^ or 10^3^ copies of target template were stopped at different time points (5, 10, 15, 20, 25, or 30 min). Generation of amplicons were detected by gel electrophoresis (Fig. 4A) followed by densitometry analysis (Fig. 4B), or by S/N ratios of fluorescent signals derived from TaqMan probes (Fig. 4C). Results of both analyses show that the amounts of amplicons derived from 10^3^ copies of target template became detectable between 10 and 15 min and reached plateau around 30 min. Signal intensity appeared to increase proportionally with time between 10 and 25 min, indicating efficient hydrolysis of TaqMan probes. In addition, compared to signals derived from10^3^ copies of target template, amplicon bands (Fig. 4, A and B) and optical signals (Fig. 4C) in reactions containing 10^1^ copies of target template became detectable at later times (about 25 min). Therefore, the optimized WSSV TaqMan probe-iiPCR, as described in Materials and Methods, included 150 nM WSSV probe and was incubated for at least 30 min.

Furthermore, for reactions using fluorescent dyes that emit basal level signals, a reliable cut-off value needs to be established to define positive and negative reactions. Therefore, whether signal-to-noise (S/N) ratios could be applied to identify positive reactions was evaluated. A threshold was tentatively formulated as follows: mean S/N ratio of non-template blank reactions +5 standard deviations (SD). The mean and SD of S/N ratios from 20 blank WSSV TaqMan probe-iiPCR reactions (Supplementary Table 1) were 1.04 and 0.06, respectively, resulting in a threshold of 1.34. Retrospective analysis of previous reactions, as represented in Figure 3 and 4, showed that S/N ratios of positive reactions containing various copies of standard plasmid DNA were all above 1.34. Moreover, all no-template control reactions produced S/N ratios below 1.34. The reliability of this threshold was evaluated further in the following experiments.

### Sensitivity Evaluation of WSSV TaqMan Probe-iiPCR

**Figure 5:**
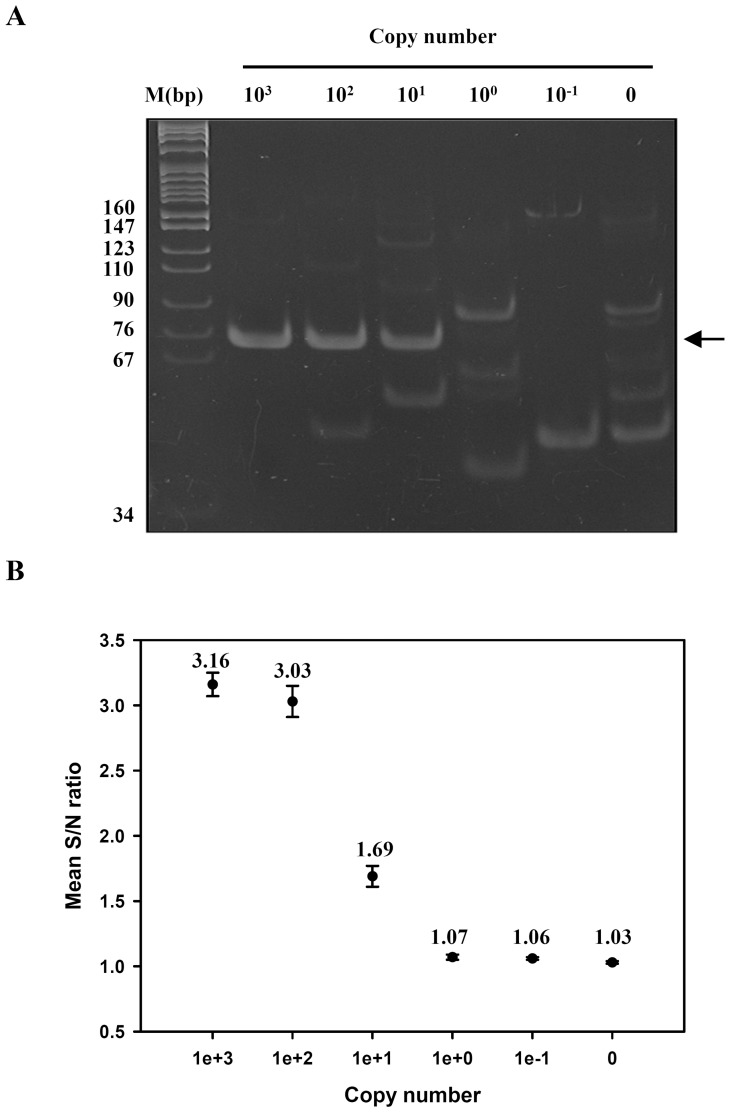
Analytical sensitivity of WSSV TaqMan probe-iiPCR. Ten-fold serial dilutions (10^3^ to 0 copies) of plasmid DNA were subjected to WSSV TaqMan probe-iiPCR amplification. Amplicons were detected by 12% polyacrylamide gel analysis (A). Fluorescent signals were collected before and after the reaction, and S/N ratios and SDs were calculated (B). Arrows, iiPCR amplicons; M, DNA size markers; “before”, fluorescent signals detected before reaction; “after”, fluorescent signals detected after reaction; S/N ratio, fluorescent intensity_after_/fluorescent intensity_before_.

First, serial dilutions of standard DNA were used to assess analytical sensitivity of the optimized WSSV TaqMan probe-iiPCR assay. An example is shown in Figure 5. In agreement with the results of gel analysis (Fig. 5A), positive S/N ratios were detected from as low as 10^1^ copies of the plasmid template (Fig. 5B). Lower S/N ratios were generated from 10^1^ copies than higher copies of the template, consistent with amplicon levels detected by gel electrophoresis. Positive signals (1.69±0.08) were consistently produced from three independent experiments containing 10^1^ copies of the plasmid template, indicating that the WSSV TaqMan probe-iiPCR assay could detect its target sensitively.

### Detection of WSSV DNA in Field Samples by WSSV TaqMan Probe-iiPCR Assay

To evaluate whether the established WSSV TaqMan probe-iiPCR assay could be applied to detect WSSV DNA in field samples, infected and uninfected L. *vannamei* samples identified previously by IQ2000 WSSV DPS, an OIE-registered commercial kit, were subjected to WSSV TaqMan probe-iiPCR assay. In one study (Table 1), the levels of WSSV DNA in the samples were determined in parallel by real-time PCR assay, for which the standard curve (R^2^ = 0.994, efficiency  = 94.0%, slope  =  −3.474) was generated by using 10-fold serial dilutions (10^4^ to 10^1^ copies) of the standard DNA. The established assay produced amplicons (data now shown) and positive S/N ratios from ∼10^1^ to 10^3^ copies of WSSV target (Table 1, samples 1–6). Samples that were designated as negative by IQ2000 WSSV DPS and real-time PCR analysis generated S/N ratios lower than the threshold in WSSV TaqMan probe-iiPCR assay (Table 1, samples 7–12).

**Table 1:** Detection of WSSV DNA in shrimp samples by IQ 2000 WSSV D&P System, WSSV TaqMan probe-based iiPCR, and real-time PCR assays.

Samples	IQ2000 WSSV DPS	TaqMan probe-based iiPCR^a^			Real-time PCR^b^ (copies/reaction)
		before	after	S/N ratio	
1	+	31.58	99.04	3.14	48
2	+	31.55	91.67	2.91	68
3	+	31.77	75.31	2.37	911
4	+	32.08	90.99	2.84	495
5	+	30.69	89.62	2.92	1240
6	+	30.09	87.73	2.92	5450
7	−	31.36	29.72	0.95	ND
8	−	30.91	29.31	0.95	ND
9	−	31.26	29.11	0.93	ND
10	−	30.06	30.13	1.00	ND
11	−	30.47	28.79	0.95	ND
12	−	30.38	28.94	0.95	ND

a Fluorescent signals in TaqMan probe-iiPCR assays were recorded before and after the reaction. S/N ratio =  signal intensity_after_/signal intensity_before_.

b The standard curve was established using serial ten-fold dilutions (10^4^ to 10^1^ copies) of standard DNA. R^2^ and slope of the WSSV real-time PCR assay were 0.994 and −3.474, respectively.

False positive rate of the established assay was further tested by analyzing 120 samples of no or low WSSV infection which were defined by IQ2000 WSSV DPS (Supplementary Table 2 and Supplementary Fig. 1). Analysis of positive cases indicated that TaqMan probe-iiPCR had 100.0% sensitivity (95% confidence interval: 94.1% to 100.0%). Furthermore, positive signals (1.37 and 1.60, Supplementary Table 2, samples 19 and 34) were generated in TaqMan probe-iiPCR from two of the 60 WSSV-negative samples, resulting in 96.7% specificity (95% confidence interval: 88.5% to 99.6%) (Table 2). The results show 96.67% agreement (Kappa test) between the two assays. Over all, results of WSSV TaqMan probe-iiPCR correlated well with those of IQ2000 WSSV DPS, implying that a reliable threshold could be established for TaqMan probe-iiPCR to achieve accurate diagnosis of pathogens in field samples.

**Table 2:** Agreement of WSSV DNA detection between IQ2000 WSSV D&P System and WSSV TaqMan probe-based iiPCR.

		TaqMan probe-based iiPCR	
		Positive	Negative
IQ2000 WSSV D&P System	Positive	60	0
	Negative	2	58

## Discussions

A thermally baffled device that protects reaction vessels from temperature fluctuations in the environment was shown previously to improve reproducibility of convective PCR in capillary tubes [21] [12]. However, the requirement of gel electrophoresis for amplicon detection has impeded further application of this method. In addition, NSPs were noted especially in reactions containing low copies or none of the target template (Figs. 1 and 5), making identification of target amplicon quite challenging. In this study, 6-FAM™-labeled/NFQ-quenched TaqMan probe was incorporated into iiPCR to generate fluorescent signals, which were detected successfully by an optical detection module integrated into the iiPCR device. Initially, WSSV and IMNV TaqMan probe-iiPCR assays were developed using plasmid DNA templates. Both assays produced significant fluorescent signals from target DNA, resulting in substantial increases in S/N ratios (signal intensity_after_/signal intensity_before_) that correlated with detection of amplicons by gel electrophoresis analysis.

**Table 3:** Time and costs for TaqMan probe-iiPCR and relevant PCR methods.

	TaqMan probe-iiPCR	Real time PCR	Nested PCR
Time^a^ (min)	35∼40	60∼120	150∼180
Cost^b^ (USD/reaction)	1∼1.5	1∼1.5	0.5∼1

a including time for iiPCR and amplicon analysis.

b based on cost of commercially available licensed reagents.

Fig. 3 shows that the S/N values increased almost linearly with concentration of TaqMan probe at lower concentrations (below 75 and 50 nM for WSSV and IMNV assays, respectively), indicating the rate of probe hydrolysis was relatively steady under these concentrations. However, increases of S/N values declined gradually at higher concentrations (Fig. 3A and 3B). Based on these results, the concentration of TaqMan probe was adjusted to 150 nM in the optimized assay.

Further evaluation of TaqMan probe-based iiPCR was focused on the WSSV assay. The threshold (mean S/N ratio of non-template blank reactions +5 SD) of WSSV TaqMan probe-assay was defined on the basis of the optical signals obtained from 20 blank reactions for classification of positive reactions (Supplementary Table 1). The obtained threshold appeared to be adequate to categorize positive and negative reactions, as S/N ratios of all WSSV-positively samples (Figs. 3, 4 and 5, and Tables 1 and 2) were higher than the calculated threshold of 1.34. Both TaqMan probe-based iiPCR assay and iiPCR/gel electrophoresis assay described earlier [12] reached similar sensitivity (10^1^ copies of WSSV DNA) in analytical and field samples (Fig. 5 and Table 1).

Furthermore, the estimated false positive rate was around 3.3% on the basis of 60 WSSV-negative samples defined by IQ2000 WSSV DPS. The two inconsistent samples generated relatively low S/N ratios (<2) in TaqMan probe-iiPCR. It is likely that these samples contained low levels of WSSV target DNA. A 3.3% false positive rate should be reasonably tolerated according to Poisson statistics. Noise contribution based on the Poisson effect results in decreases in assay precision below 1000 copies of target DNA. The accuracy and reliability of the threshold needs to be further assessed and validated with more field samples. Certainly, for each TaqMan probe-iiPCR, reliable threshold S/N ratios should be defined empirically by evaluating large numbers of positive and blank reactions.

In our hands, NSPs appeared to be produced more frequently in iiPCR than in convectional PCR. It is likely due to that that iiPCR cycles are carried out under less defined conditions than conventional PCR to allow hybridization of primers to off-target sequences. Therefore, dsDNA intercalating dyes (such as SYBR Green I), which are not capable of differentiating target amplicons from NSPs, were not used in this study even though they are less expensive than TaqMan probes. On the contrary, NSPs generally do not result in false positive signals in a TaqMan probe-based assay because specificity of the probe makes it unlikely to produce signals from irrelevant products. TaqMan probes designed according to the rules for real-time PCR have rarely generated S/N ratios significantly higher than 1.0 in iiPCR reaction generating only NSPs (Table 1 and Supplementary Table 2). However, contamination of NSPs could impede production of target amplicons, resulting in reduction in sensitivity. Therefore, procedures to prevent cross contamination of iiPCR products should be implemented stringently.

The convection driven by temperature gradients within the reaction vessel facilitates rapid cycles of the denaturation, annealing, and extension steps in convective PCR [9], [10], [22]. Krishnan’s model of convective PCR requires approximately 20 to 25 sec to complete one cycle [22], finishing 36 to 45 cycles within 15 min theoretically. In TaqMan probe-iiPCR, the minimal time required to generate detectable positive signals from 10^3^ copies of target DNA was between 10 and 15 min (Fig. 4). After 20 min, the levels of amplicons detected by gel electrophoresis appeared to reach plateau but the S/N ratios continued to increase at relatively slower rates (Fig. 4A, 10^3^ copies). Compared to gel electrophoresis, the TaqMan probe hydrolysis/optical detection system has wider dynamic ranges of signal detection for iiPCR. Furthermore, 10^1^-copies reactions required longer incubator times than 10^3^-copy reactions to generate detectable signals, which was also able to rise progressively and effectively afterwards. The results suggest that a 30-min incubation period would ensure accumulation of significant probe signals in reactions containing low copies of target.

The optical detection module, capable of emitting blue LED light through a 505-nm short bandpass filter and collecting fluorescent signals with a 520-nm bandpass filter, was designed to detect fluorescent signals from 6-FAM™. Principally, the 6-FAM™ reporter dye could be replaced by other dyes of similar excitation and emission spectra, such as fluorescein, FITC, and Alexa Fluor 488, and DyLight 488. The signal-to-noise (S/N) ratio was calculated by dividing fluorescent signal (after reaction) with basal signal (before reaction). Consequently, lower basal signal intensity would lead to higher S/N ratios. The efficiency of a quencher depends on the physical distance between the quencher and reporter moiety in a probe [23]. Probes should be as short as possible to bring the quencher and the reporter close to each other in three dimensions. Labeling the probe with NFQ quencher dyes could also help decrease basal signal levels since NFQs do not emit any fluorescence. NFQ dyes, such as QSY-7 (maximum excitation wavelength 560 nM) and QAY-9 (maximum excitation wavelength 562 nM), are suitable candidates for quencher dye.

Signal production from TaqMan probes relies on efficient probe hydrolysis by the 5′ exonuclease activity of Taq DNA polymerase at the extension step of PCR [24]. Several commercial Taq DNA polymerases, including TaKaRa Taq™ (Takara Bio, Shiga, Japan), Taq DNA Polymerase (New England Biolabs, Ipswich, MA.), and GoTaq® Flexi DNA Polymerase (Promega, Madison, WI) were tested and appropriate signals were detected in iiPCR (data not shown). We have noted that increases of iiPCR products (estimated by gel electrophoresis analysis) did not always result in elevation of S/N ratios derived from TaqMan probe. One possible explanation is that the TaqMan probe hybridizes to target DNA less efficiently than iiPCR primers, leading to failure of probe-target binding before primer extension occurs. Probes that meet criteria such as appropriate Tm and no stable secondary structure should ensure sufficient probe hybridization to the template at the extension step. Strategies to enhance probe performance include conjugation of minor groove binding (MGB) group to its 3′-end [25] and incorporation of locked nucleic acids (LNA) into the probe [26].

In this study, we demonstrated that it is possible to define a threshold value of end-point S/N ratios to differentiate positive and negative samples. Although the time taken to accumulate detectable signals and the amounts of input template appeared to correlate negatively with each other, more detailed analyses are required to clarify whether a linear correlation exists between these two parameters for possible development of quantitative TaqMan probe-iiPCR (Fig. 5). Quantitative TaqMan probe-iiPCR might have a much narrower linear dynamic range of detection than real-time PCR because iiPCR cycles proceed at much faster rates than convectional PCR. Signals generated from high copy numbers of target DNA would likely become detectable at around similar time points.

Using disposable reaction vessels and requiring no post-amplification processing TaqMan probe-iiPCR optical detection method should offer a promising rapid and low-cost platform for on-site diagnosis of diseases. In comparison with methods that offer similar sensitivity and specificity, iiPCR should take less time to complete than conventional PCR methods (Table 3), which require ramping time between different steps. The costs of reagents for both iiPCR and real-time PCR should be similar because both assays use similar reagents for amplification and signal generation. However, the device for iiPCR would be much simpler and cost much less than real-time PCR machines. Requiring not fluorescent dye-labeled probe, nested PCR costs less than iiPCR (Table 3). Nevertheless, post-amplification processing (such as gel electrophoresis) could pose serious problem.

In conclusion, results of this study support that convective iiPCR, TaqMan probe hydrolysis and an optical detection module have been integrated successfully to achieve highly sensitive detection of the target pathogen. Using disposable reaction vessels and requiring no post-amplification processing, the TaqMan probe-iiPCR optical detection method should offer a promising low-cost platform for sensitive, specific and rapid on-site diagnosis of diseases. The optical setup described in this report was designed for the detection of only one reporter dye. A system that can detect two reporter dyes is currently being developed to allow the inclusion of an internal control for nucleic acid extraction and amplification steps.

## Supporting Information

Figure S1 **Determination of shrimp samples by IQ2000 WSSV Detection and Prevention System.** Genomic DNA extractions of shrimps were subjected to amplification by IQ2000 WSSV DPS. Positive standard DNAs provided by the IQ2000 kits were diluted (10^3^, 10^2^, 10^1^ copies) and included in the assays. The IQ2000 kits were designed to generate three amplicons (closed arrow heads) from the target sequences. The number of the product bands correlates positively with the starting concentrations of target DNA. In addition, the presence of the internal-control signal (open arrow heads) and the absence of target signals implicate that the samples are target-pathogen free. M, DNA size markers; N, water only.
(PDF)

Table S1 **Analysis results of non-template samples by WSSV TaqMan probe-based iiPCR assay.**
(DOC)

Table S2 **Analysis results of WSSV negative and positive samples by WSSV TaqMan probe-based iiPCR assay.**
(DOC)
