# Supplementary figures and images for: Development of TaqMan Probe-Based Insulated Isothermal PCR (iiPCR) for Sensitive and Specific On-Site Pathogen Detection
Source: PLoS One. 2012 Sep 25;7(9):e45278. doi: 10.1371/journal.pone.0045278 (PMC3458002; doi:10.1371/journal.pone.0045278)

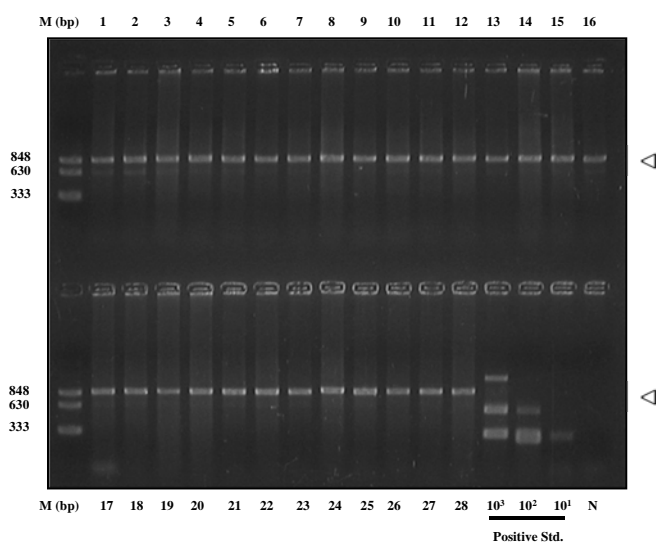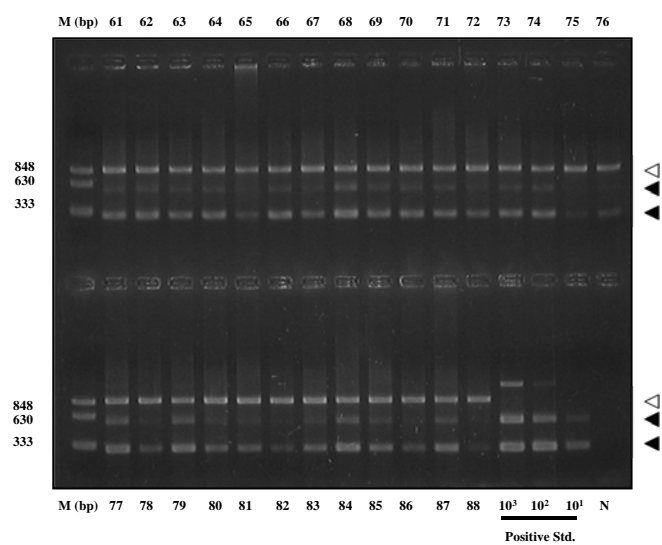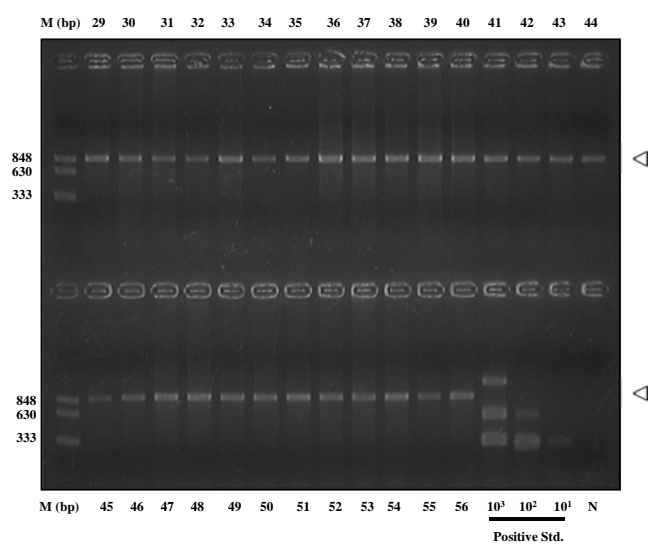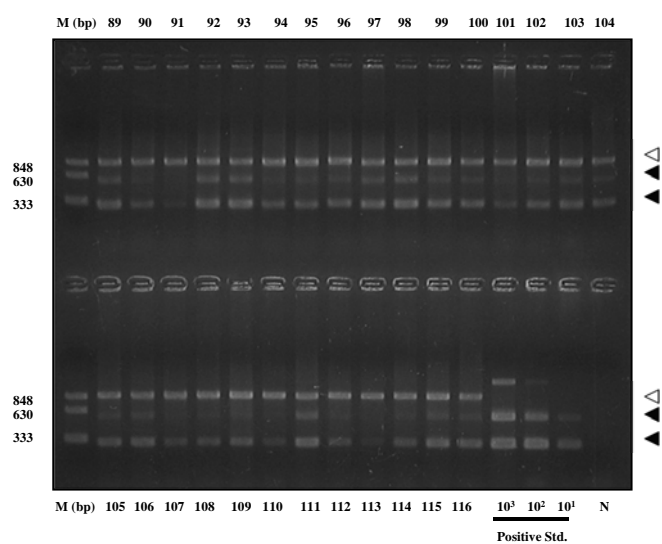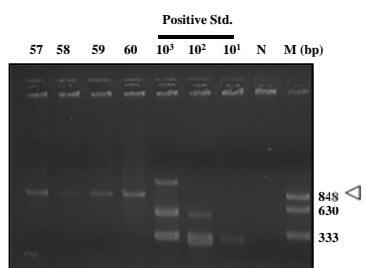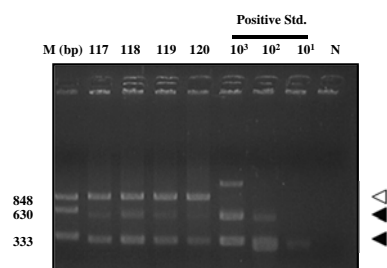

Supplement: Figure S1 — Determination of shrimp samples by IQ2000 WSSV Detection and Prevention System. Genomic DNA extractions of shrimps were subjected to amplification by IQ2000 WSSV DPS. Positive standard DNAs provided by the IQ2000 kits were diluted (103, 102, 101 copies) and included in the assays. The IQ2000 kits were designed to generate three amplicons (closed arrow heads) from the target sequences. The number of the product bands correlates positively with the starting concentrations of target DNA. In addition, the presence of the internal-control signal (open arrow heads) and the absence of target signals implicate that the samples are target-pathogen free. M, DNA size markers; N, water only. (PDF) [file pone.0045278.s001.pdf]
