# Supplementary material for: Development of TaqMan Probe-Based Insulated Isothermal PCR (iiPCR) for Sensitive and Specific On-Site Pathogen Detection
Source: PLoS One. 2012 Sep 25;7(9):e45278. doi: 10.1371/journal.pone.0045278 (PMC3458002; doi:10.1371/journal.pone.0045278)
Supplement: Table S1 — Analysis results of non-template samples by WSSV TaqMan probe-based iiPCR assay. (DOC) [file pone.0045278.s002.doc]

**Table S1. Analysis results of non-template samples by WSSV TaqMan probe-based iiPCR assay**

| **Sample** | **1** | **2** | **3** | **4** | **5** | **6** | **7** | **8** | **9** | **10** | **11** | **12** | **13** | **14** | **15** | **16** | **17** | **18** | **19** | **20** |
| --- | --- | --- | --- | --- | --- | --- | --- | --- | --- | --- | --- | --- | --- | --- | --- | --- | --- | --- | --- | --- |
| **B520** | **31.66** | **31.41** | **31.17** | **30.44** | **30.38** | **31.53** | **31.21** | **30.40** | **31.12** | **30.36** | **32.02** | **30.85** | **30.63** | **30.63** | **30.64** | **30.40** | **30.92** | **30.01** | **30.83** | **30.41** |
| **A520** | **35.04** | **28.70** | **31.27** | **27.70** | **27.38** | **32.47** | **32.11** | **35.06** | **33.47** | **31.71** | **33.87** | **32.16** | **32.88** | **33.43** | **32.35** | **31.95** | **32.71** | **32.41** | **33.22** | **32.95** |
| **S/N** | **1.11** | **0.91** | **1.00** | **0.91** | **0.90** | **1.03** | **1.03** | **1.15** | **1.08** | **1.04** | **1.06** | **1.04** | **1.07** | **1.09** | **1.06** | **1.05** | **1.06** | **1.08** | **1.08** | **1.08** |
