# Supplementary material for: Development of TaqMan Probe-Based Insulated Isothermal PCR (iiPCR) for Sensitive and Specific On-Site Pathogen Detection
Source: PLoS One. 2012 Sep 25;7(9):e45278. doi: 10.1371/journal.pone.0045278 (PMC3458002; doi:10.1371/journal.pone.0045278)
Supplement: Table S2 — Analysis results of WSSV negative and positive samples by WSSV TaqMan probe-based iiPCR assay. (DOC) [file pone.0045278.s003.doc]

**Table S2. Analysis results of WSSV negative and positive samples by WSSV TaqMan probe-based iiPCR assay**

| **Sample** | **1** | **2** | **3** | **4** | **5** | **6** | **7** | **8** | **9** | **10** | **11** | **12** | **13** | **14** | **15** | **16** | **17** | **18** | **19** | **20** |
| --- | --- | --- | --- | --- | --- | --- | --- | --- | --- | --- | --- | --- | --- | --- | --- | --- | --- | --- | --- | --- |
| **B520** | **30.15** | **31.06** | **33.14** | **32.81** | **31.52** | **31.86** | **32.06** | **30.75** | **30.73** | **31.21** | **32.43** | **31.46** | **32.38** | **30.79** | **30.79** | **31.46** | **31.47** | **31.51** | **32.63** | **32.63** |
| **A520** | **36.17** | **33.03** | **32.98** | **33.92** | **33.55** | **33.59** | **32.63** | **28.73** | **31.70** | **34.24** | **34.71** | **34.96** | **35.55** | **34.35** | **29.66** | **34.73** | **32.95** | **30.74** | **52.33** | **34.92** |
| **S/N** | **1.20** | **1.06** | **1.00** | **1.03** | **1.06** | **1.05** | **1.02** | **0.93** | **1.03** | **1.10** | **1.07** | **1.11** | **1.10** | **1.12** | **0.96** | **1.10** | **1.05** | **0.98** | **1.60** | **1.07** |

| **Sample** | **21** | **22** | **23** | **24** | **25** | **26** | **27** | **28** | **29** | **30** | **31** | **32** | **33** | **34** | **35** | **36** | **37** | **38** | **39** | **40** |
| --- | --- | --- | --- | --- | --- | --- | --- | --- | --- | --- | --- | --- | --- | --- | --- | --- | --- | --- | --- | --- |
| **B520** | **30.80** | **31.66** | **32.24** | **31.95** | **32.90** | **31.01** | **30.31** | **30.05** | **30.59** | **33.29** | **32.47** | **30.65** | **31.20** | **30.86** | **30.92** | **31.36** | **30.84** | **31.95** | **30.15** | **30.02** |
| **A520** | **32.84** | **33.40** | **34.45** | **34.32** | **34.86** | **33.17** | **32.63** | **32.04** | **32.42** | **35.32** | **33.95** | **32.78** | **33.05** | **42.38** | **32.36** | **31.90** | **27.90** | **33.39** | **31.92** | **32.19** |
| **S/N** | **1.07** | **1.05** | **1.07** | **1.07** | **1.06** | **1.07** | **1.08** | **1.07** | **1.06** | **1.06** | **1.05** | **1.07** | **1.06** | **1.37** | **1.05** | **1.02** | **0.90** | **1.05** | **1.06** | **1.07** |

| **Sample** | **41** | **42** | **43** | **44** | **45** | **46** | **47** | **48** | **49** | **50** | **51** | **52** | **53** | **54** | **55** | **56** | **57** | **58** | **59** | **60** |
| --- | --- | --- | --- | --- | --- | --- | --- | --- | --- | --- | --- | --- | --- | --- | --- | --- | --- | --- | --- | --- |
| **B520** | **31.21** | **32.11** | **31.56** | **31.56** | **32.39** | **30.06** | **30.84** | **32.12** | **30.70** | **32.42** | **31.87** | **31.77** | **30.51** | **31.42** | **31.38** | **31.11** | **30.97** | **30.68** | **32.57** | **33.20** |
| **A520** | **30.83** | **33.02** | **29.89** | **32.97** | **33.30** | **32.54** | **31.39** | **33.46** | **29.93** | **33.28** | **31.29** | **31.50** | **33.09** | **32.23** | **31.31** | **32.29** | **30.38** | **32.90** | **31.26** | **32.44** |
| **S/N** | **0.99** | **1.03** | **0.95** | **1.04** | **1.03** | **1.08** | **1.02** | **1.04** | **0.97** | **1.03** | **0.98** | **0.99** | **1.08** | **1.03** | **1.00** | **1.04** | **0.98** | **1.07** | **0.96** | **0.98** |

| **Sample** | **61** | **62** | **63** | **64** | **65** | **66** | **67** | **68** | **69** | **70** | **71** | **72** | **73** | **74** | **75** | **76** | **77** | **78** | **79** | **80** |
| --- | --- | --- | --- | --- | --- | --- | --- | --- | --- | --- | --- | --- | --- | --- | --- | --- | --- | --- | --- | --- |
| **B520** | **30.87** | **32.35** | **31.76** | **32.98** | **31.59** | **31.11** | **32.67** | **32.08** | **31.12** | **32.05** | **30.39** | **30.35** | **30.74** | **32.40** | **30.94** | **30.04** | **32.32** | **32.63** | **31.68** | **31.35** |
| **A520** | **77.13** | **69.45** | **49.91** | **61.78** | **44.77** | **56.68** | **62.19** | **79.10** | **85.60** | **64.90** | **83.96** | **46.54** | **51.08** | **94.64** | **93.77** | **61.05** | **91.11** | **86.07** | **86.72** | **72.56** |
| **S/N** | **2.50** | **2.15** | **1.57** | **1.87** | **1.42** | **1.82** | **1.90** | **2.47** | **2.75** | **2.02** | **2.76** | **1.53** | **1.66** | **2.92** | **3.03** | **2.03** | **2.82** | **2.64** | **2.74** | **2.31** |

| **Sample** | **81** | **82** | **83** | **84** | **85** | **86** | **87** | **88** | **89** | **90** | **91** | **92** | **93** | **94** | **95** | **96** | **97** | **98** | **99** | **100** |
| --- | --- | --- | --- | --- | --- | --- | --- | --- | --- | --- | --- | --- | --- | --- | --- | --- | --- | --- | --- | --- |
| **B520** | **30.60** | **31.87** | **31.40** | **31.07** | **30.23** | **32.02** | **30.82** | **33.43** | **32.75** | **31.72** | **30.46** | **30.56** | **30.20** | **32.79** | **31.38** | **32.69** | **31.68** | **31.36** | **32.10** | **30.55** |
| **A520** | **60.34** | **61.69** | **89.49** | **81.14** | **45.93** | **89.77** | **58.62** | **66.83** | **54.90** | **72.50** | **54.75** | **86.07** | **84.43** | **75.14** | **70.55** | **61.69** | **74.43** | **86.89** | **63.07** | **67.68** |
| **S/N** | **1.97** | **1.94** | **2.85** | **2.61** | **1.52** | **2.80** | **1.90** | **2.00** | **1.68** | **2.29** | **1.80** | **2.82** | **2.80** | **2.29** | **2.25** | **1.89** | **2.35** | **2.77** | **1.97** | **2.22** |

| **Sample** | **101** | **102** | **103** | **104** | **105** | **106** | **107** | **108** | **109** | **110** | **111** | **112** | **113** | **114** | **115** | **116** | **117** | **118** | **119** | **120** |
| --- | --- | --- | --- | --- | --- | --- | --- | --- | --- | --- | --- | --- | --- | --- | --- | --- | --- | --- | --- | --- |
| **B520** | **32.07** | **31.49** | **30.58** | **31.92** | **30.68** | **31.94** | **33.27** | **30.94** | **30.56** | **31.35** | **31.54** | **32.76** | **30.48** | **30.37** | **33.45** | **30.63** | **31.80** | **33.24** | **31.19** | **30.98** |
| **A520** | **65.39** | **79.80** | **67.39** | **89.91** | **89.07** | **84.46** | **77.97** | **84.02** | **66.91** | **83.29** | **60.29** | **90.98** | **89.57** | **59.88** | **99.57** | **65.06** | **85.19** | **88.74** | **80.60** | **57.80** |
| **S/N** | **2.04** | **2.53** | **2.20** | **2.82** | **2.90** | **2.64** | **2.34** | **2.72** | **2.19** | **2.66** | **1.91** | **2.78** | **2.94** | **1.97** | **2.98** | **2.12** | **2.68** | **2.67** | **2.58** | **1.87** |
